# SOCIOECONOMIC GRADIENTS IN DEPRESSIVE SYMPTOMS AMONG OLDER ADULTS IN RURAL SOUTH AFRICA

**DOI:** 10.1093/geroni/igad104.3054

**Published:** 2023-12-21

**Authors:** Mansi Verma, Lindsay Kobayashi

**Affiliations:** University of South Carolina Arnold School of Public Health, Columbia, South Carolina, United States; University of Michigan School of Public Health, Ann Arbor, Michigan, United States

## Abstract

Depression is one of the most common mental health disorders throughout the globe with an estimated 300 million people affected worldwide. Socioeconomic status may influence depression risk, due to its status as a critical influencing factor for health-related issues as well as serving as a barrier for healthy aging. The objective of this analysis was to examine the presence of socioeconomic gradients in depressive symptoms among older adults in Agincourt, a low-income rural region of South Africa. We conducted a cross-sectional analysis of baseline interview data from 2014/2015 for 5,059 participants aged ≥40 in the population-representative Health and Aging in Africa: A Longitudinal Study of an INDEPTH Community in South Africa (HAALSI). Household wealth was assessed as quintiles of an asset-based index, including ownership of assets such as housing materials, electricity, piped water, vehicles, and technological goods. Household per capita consumption on food, healthcare, leisure activities, and other goods was also assessed as quintiles. The presence of depressive symptoms was measured and categorized using the 8-item Center for Epidemiologic Studies Depression (CES-D) scale. We used multivariable adjusted logistic regression to examine the association between household wealth and household consumption quintiles and depression. In this study population, the prevalence of depressive symptoms was 17%. We observed no significant trends between household wealth, household consumption and depression (p=0.11 for both exposures). Depressive symptoms are not socioeconomically graded among older, rural South Africa adults. The high prevalence of depressive symptoms within the region should be considered as a public health concern.

